# Chronotype as a potential risk factor for cognitive decline: The mediating role of sleep quality and health behaviours in a 10-year follow-up study

**DOI:** 10.1016/j.tjpad.2025.100168

**Published:** 2025-04-11

**Authors:** A.N. Wenzler, A.C. Liefbroer, R.C. Oude Voshaar, N. Smidt

**Affiliations:** a Department of Epidemiology, University of Groningen, University Medical Center Groningen, FA40 PO30.001, Groningen 9700 RB, the Netherlands; b Netherlands Interdisciplinary Demographic Institute (NIDI)–Royal Netherlands Academy of Sciences (KNAW), Lange Houtstraat 19, 2511 CV, The Hague, the Netherlands; c Department of Sociology, Vrije Universiteit Amsterdam (VU), Amsterdam, the Netherlands; d Department of Psychiatry, University of Groningen, University Medical Center Groningen, Groningen, the Netherlands

**Keywords:** Chronotype, Cognitive decline, Sleep quality, Health behaviours

## Abstract

**Background:**

– With rising life expectancies and ageing populations worldwide, preserving cognitive health is an urgent global priority. Chronotype could be a potential risk factor for cognitive decline, potentially through mediators sleep quality, alcohol intake, physical activity, and smoking.

**Methods:**

– This study used data from participants aged 40 years and older from the Lifelines cohort study (*n* = 23,798). Chronotype, assessed with the Munich ChronoType Questionnaire, was included as a continuous score of mid-point sleep corrected for sleep debt on workdays. Multiple linear regression examined the association between chronotype and cognitive decline, including moderation by age, educational attainment, and sex. The KHB-method was applied to test mediation by sleep quality, alcohol intake, physical activity, and smoking.

**Outcomes:**

– Cognition was assessed with the Ruff Figural Fluency Test (RFFT), measuring non-verbal fluency and executive functioning. Cognitive decline was calculated by subtracting the RFFT sum score at baseline from the 10-year follow-up score.

**Results:**

– Chronotype was associated with cognitive decline. Educational attainment, but not age or sex, moderated the relationship. No significant associations were observed in the low- (0.07, 95 % CI: -0.44, 0.57) or middle- (-0.41, 95 % CI: -0.88, 0.06) educational groups. In the high-educational group each one-hour increase in chronotype corresponded to a 0.80-point decline in cognition per decade (95 % CI: -1.34, -0.26). In this group, sleep quality and current smoking mediated 13.52 % and 18.64 % of the association, respectively.

**Interpretation:**

– Chronotype was associated with greater decline in non-verbal fluency and executive functioning among higher educated participants, highlighting the importance of targeted prevention strategies.

**Funding:**

– This work is part of the BIRD-NL consortium funded by the Dutch Medical Research Council, ZonMw (Dementia research program) project number:10,510,032,120,005.

## 1 Introduction

Population ageing has led to an increase in dementia with currently 55 million cases worldwide, which is expected to increase to 132 million cases by 2050. About 45 % of dementia cases can be attributed to twelve modifiable risk factors, while the remaining 55 % are attributed to age, sex, genetics, and currently unknown factors [1]. Dementia has a long preclinical phase, with pathological changes starting in midlife, followed by years of accelerated cognitive decline before diagnosis [2]. One potential novel risk factor for cognitive decline is chronotype, more commonly known as being an “early lark” or a “night owl” [3]. Chronotype reflects individual preferences for activity and sleep times, regulated by the circadian rhythm, a 24 h cycle regulating various physiological processes (e.g., body temperature, sleeping patterns, and hormone secretion) [4]. During puberty, individuals shift from an earlier to a later chronotype; later, during midlife, they shift back to an earlier chronotype [5]. However, this is not the case for all individuals. Around 7.1 % of adults have an (extreme) early chronotype and 19.9 % have an (extreme) late chronotype at midlife [6]. While dim light melatonin onset is considered the most reliable chronotype measure [4], it is rarely used in epidemiological cohort studies due to being time-consuming, expensive and burdensome for people [7]. Instead, self-report questionnaires, such as the Munich ChronoType Questionnaire (MCTQ), are commonly used [4,8]. The MCTQ bases chronotype on sleep and wake time during working and non-working days by calculating Mid-point Sleep on Free days corrected for sleep debt on workdays (MSF_SC_). MSF_SC_ is a continuous measure of chronotype ranging from extremely early (< 12:00 AM) to extremely late chronotypes (> 9:00 AM).

Due to societal demands (e.g., job starting times, social gatherings and children) the schedules of individuals may not align with their chronotype [4], leading to “social jetlag” [8]. Early chronotypes, who peak in alertness early in the day, often stay awake into their biological night due to evening social commitments, which can reduce sleep duration and quality [8]. Adjusting their life to their early chronotype may limit social activities such as for example physical activity, as this takes place mostly during the evening hours. Conversely, evening chronotypes experience energy later in the day, making it difficult to fall asleep at typical times, leading to challenges waking up for morning schedules [8]. Evening chronotypes also tend to report poorer sleep quality [9] and have been linked to unhealthy lifestyle behaviours such as alcohol intake [10], smoking [11] and physical inactivity [12], as well as higher risk of obesity [13], type II diabetes [14], and neurodegenerative disorders [15]. However, is important to note that some associations between a late chronotype and negative health outcomes (e.g., cardiovascular disease) also exist irrespectively of the presence of social jetlag [16].

A disrupted circadian rhythm has been associated with dementia pathology [17]. Circadian disruptions, which can results from shift work [18] or frequently experiencing jet lag [19], may contribute to neurodegeneration[20]; however, neurodegeneration in the suprachiasmatic nucleus can also lead to disruption in the circadian rhytm [15], suggesting a bidirectional relationship [20]. Chronotype reflects milder variations in circadian rhythm disruptions (e.g., being an early lark or a night owl), but whether these differences are associated with dementia remains unclear. The exact mechanism linking chronotype to cognitive decline are not yet fully understood. However, given the known links between more extreme circadian rhythm disruption and dementia, it is plausible that similar mechanisms contribute to the association between chronotype and cognitive decline. Several hypotheses have been proposed to explain this relationship. Firstly, the circadian clock may play a key role in regulating the expression of neuroprotective proteins [17], and prolonged disruptions can overwhelm this protective signalling thereby leading to neuron loss. Secondly, the circadian clock could also play a role in blood-brain barrier permeability, which has implications for protein clearance from the brain [21]. However, current evidence for this is primarily based on animal models. Thirdly, circadian rhythm disruptions could alter sleep behaviours, raising the amyloid-beta (Aβ) and tau burden in the brain [22]. Poorer sleep quality is associated with increased Aβ in Alzheimer sensitive brain regions [23], leading to decline in overall cognition [24] and decline in specific cognitive domains such as executive functioning [25], although these effects are small. Finally, individuals with a late chronotype more often have unhealthy behaviours such as higher alcohol intake [10], physical inactivity [12], and smoking [11]. These unhealthy behaviours have been associated with cognitive decline and dementia [1]. To date, only a few longitudinal cohort studies have investigated the association between chronotype and cognitive decline. The results of these studies are inconsistent, probably due to differences in operationalisation of chronotype (e.g., defining early chronotype as <1:00 [27], <2:16 [26] or basing it on polygenetic risk scores [28]), types of outcome measures, timing of outcome assessment and study populations [[26], [27], [28]].

The primary aim of this study is to investigate the longitudinal association between chronotype and cognitive decline among adults of the general population aged 40 years and older. We hypothesise a U-shaped association, whereby individuals with more extreme early chronotypes or late chronotypes will experience greater cognitive decline compared to those having an intermediate chronotype. Previous research has demonstrated that women, older individuals and those with lower educational attainment have a higher risk of developing dementia [29,30]. Therefore, we will investigate whether the association between chronotype and cognitive decline is moderated by age, educational attainment and sex. Finally, to understand the potential pathways, we will investigate whether sleep quality, alcohol intake, physical activity and smoking mediate the association between chronotype and cognitive decline. These variables are included as mediators given the established effect of chronotype on sleep quality [9] and these health behaviours [[10], [11], [12]], as well as the association between sleep quality and these health behaviours and cognitive decline [1]. We hypothesise that poor sleep quality, high alcohol intake, low physical activity and current smoking will partially mediate the association between chronotype and cognitive decline among middle-aged and older adults.

## 2 Methods

### 2.1 Study design and population

**Fig. 1:**
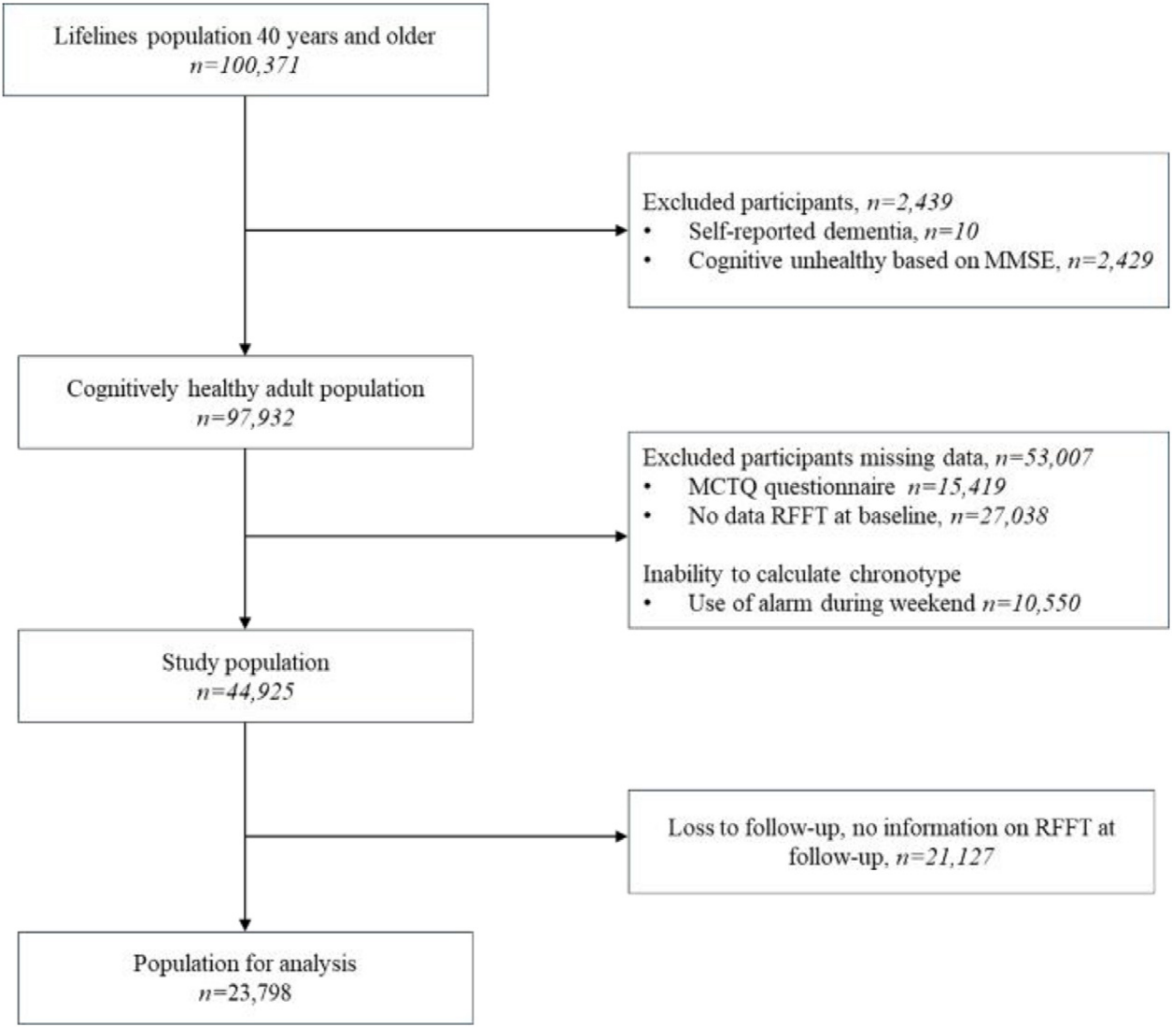
Flowchart of study participants.

Lifelines is a multi-disciplinary prospective population-based cohort study examining in a unique three-generation design the health and health-related behaviours of 167,729 persons living in the North of the Netherlands. It employs a broad range of investigative procedures in assessing the biomedical, socio-demographic, behavioural, physical and psychological factors which contribute to the health and disease of the general population, with a special focus on multi-morbidity and complex genetics, as extensively described elsewhere [31]. The Lifelines Cohort is conducted in line with the Declaration of Helsinki and received approval by the Medical Ethics Committee of the University Medical Centre Groningen (approval number: 2007/152). All included participants signed informed consent. For this study, all 100,371 participants aged 40 years and older were initially included. To select a cognitive healthy study population at baseline, we excluded participants with self-reported dementia (*n* = 10) and those with low cognitive status, defined as a Mini-Mental State Examination (MMSE) score < 26 (*n* = 2249) resulting in a sample of 97,932 cognitively healthy participants. Participants for whom chronotype at baseline could not be estimated were excluded (i.e., missing data on the Munich ChronoType Questionnaire (MCTQ) (*n* = 15,419) - the tool used to calculate chronotype - or those who used an alarm clock during the weekend (*n* = 10,550)). Participants with missing data on the outcome (i.e., cognition measured by the Ruff Figural Fluency Test at baseline (*n* = 27,038), as well as those who were lost to follow-up (*n* = 21,127)) were also excluded. This resulted in a final sample of 23,798 for the analysis (Fig. 1).

### 2.2 Data collection

Cognition was assessed using the Ruff Figural Fluency Test (RFFT), which measures nonverbal fluency and executive functioning. The current study did not include other aspects of cognition (i.e., memory or complex attention) as no data for these cognitive domains were available. The RFFT measures the ability to produce figures on five different dot configurations, with the goal to draw as many unique figures as possible [32]. The sum score (range 0 – 175) is a reliable and valid measure to capture executive functioning of an individual [33]. The RFFT was administered as a paper-pencil test at baseline and as a digital test at follow-up. Despite the change in administration method, the RFFT maintained its strong discriminatory ability [34]. Additionally, the transition from paper-pencil to digital administration had a consistent effect across participants [34]. Cognitive change over the follow-up period was calculated by subtracting the sum score on the RFFT at baseline from the sum score at follow-up.

Chronotype was assessed at baseline with the Munich ChronoType Questionnaire (MCTQ). The MCTQ has been previously validated in the Dutch adult population for assessing chronotype [35]. The MCTQ focusses on sleep habits during workdays and free (i.e., non-working) days for assessing chronotype. Chronotype was included as a continuous score of the hour of Mid-point Sleep on Free days corrected for sleep debt on workdays (MSF_SC_). This correction is essential, as not accounting for sleep debt would overestimate eveningness [36]. MSF_SC_ was included as a continuous variable in the main analysis since the category limits for defining chronotype are arbitrary [48]. Each one-hour increase in mid-point of sleep (MSF_SC_) reflects to a one-hour shift in chronotype, which ranges from extremely early to extremely late. Since calculating MSF_SC_ requires participants to wake up naturally on weekend, those who used an alarm clock during the weekend were excluded (Fig. 1).

Potential confounders and moderators included age, educational attainment, baseline RFFT score and sex. Baseline age, in years, was included as a continuous variable. Educational attainment was based on the question: *What is the highest level of education you have finished?*, and was split into three categories: low (junior general secondary education or lower), middle (secondary vocational education, work-based learning, senior general secondary education or pre-university secondary education) and high (higher vocational education or higher)[37]. Sex was included as a binary variable: female or male. Alcohol intake, physical activity, sleep quality and smoking were included as mediators. Alcohol intake was assessed using a Food Frequency Questionnaire (FFQ) with the help of two questions: 1) *How often did you drink alcoholic drinks in the past month?* 2) *How many glasses of alcoholic drinks did you drink per occasion on average?* [38]. Based on these two questions, an average daily alcohol intake was calculated. Alcohol consumption was included as a categorical variable: abstainer, light alcohol intake or heavy alcohol intake. Participants who answered “never” on the first question, were classified as abstainers. Participants with an average intake of ≤ 3 glasses of alcohol per day were classified as light alcohol intake. Participants who had an average alcohol intake of more than 3 glasses a day were classified as heavy alcohol intake [39]. Physical activity was assessed by a validated measurement tool [40], the Short Questionnaire to Assess Health-enhancing physical activity (SQUASH) questionnaire. Habitual physical activity was measured with asking how many days on average per week, how long in hours and minutes and how intense (light, moderate or vigorous) participants participated in the following activities: commuting, leisure time, household activities and work and school. Based on these questions the activity levels in minutes per week were calculated, with MET scores of ≥ 4 and < 6.5 indicating moderate and ≥ 6.5 indicating vigorously intensive activities [41,42]. Physical activity was included in hours of moderate to vigorous physical activity per week. Sleep quality was based on the Pittsburgh Sleep Quality Index (PSQI) questionnaire, a validated questionnaire [43], which consists of several questions assessing sleep duration, sleep disturbances and overall quality. The quality index score is calculated based on seven components, creating a continuous score ranging between 0 and 21, with higher scores indicating poorer sleep quality [44]. Smoking was included as a categorical variable based on two questions: 1) *Do you smoke now, or have you smoked in the past month?* 2) *Have you ever smoked a full year?* [45]. Answers to these questions led to the following categories: current smoker (question 1 = yes), past smoker (question 1= no, question 2 = yes), never smoker (question 1 and 2 = no). All variables will be presented as number and percentages (n, %) in case of categorical values. In case of continuous variables they will be presented by their mean and standard deviation (SD) when normally distributed and by median and inter quartile range (IQR) when non-normally distributed.

### 2.3 Statistical analysis

Descriptive statistics were used to describe baseline characteristics. T-tests, Wilcoxon signed-rank tests, and Chi-Square tests were used to compare baseline characteristics across educational attainment subgroups (low *n* = 6,822; middle *n* = 9165; high *n* = 7,811) and between the final population for analysis (*n* = 23,798) and those lost to follow-up (*n* = 21,127). Missing data were imputed using Multivariate Imputation by Chained Equations (MICE), with five datasets and 50 iterations each. Variables included in the imputation model were age, alcohol intake, baseline and follow-up RFFT scores, educational attainment, physical activity, sex, sleep quality, and smoking status. For the analytical sample, the following values were imputed: alcohol intake (*n* = 1,954), educational attainment (*n* = 375), physical activity (*n* = 1,603), sleep quality (*n* = 457) and smoking (*n* = 145).

Multivariable linear regression examined the association between chronotype and cognitive decline. First a non-linear U-shape between chronotype and cognitive decline was examined by including a quadratic term. If the quadratic model did not fit the data, a linear model was used. Model 1 examined the univariable association and Model 2 adjusted for age, sex and baseline RFFT score. Exact p-values were presented, with a *p* < 0.05 deemed statistically significant. Effect modification by age, sex, and educational attainment was assessed by adding an interaction term to the adjusted model (chronotype*age/sex/educational status). If an interaction for age, sex or educational attainment had a p-value of *p* < 0.10 in the multivariable model, stratified analyses were performed. Subsequently, also stratified mediation analysis was performed.

**Fig. 2:**
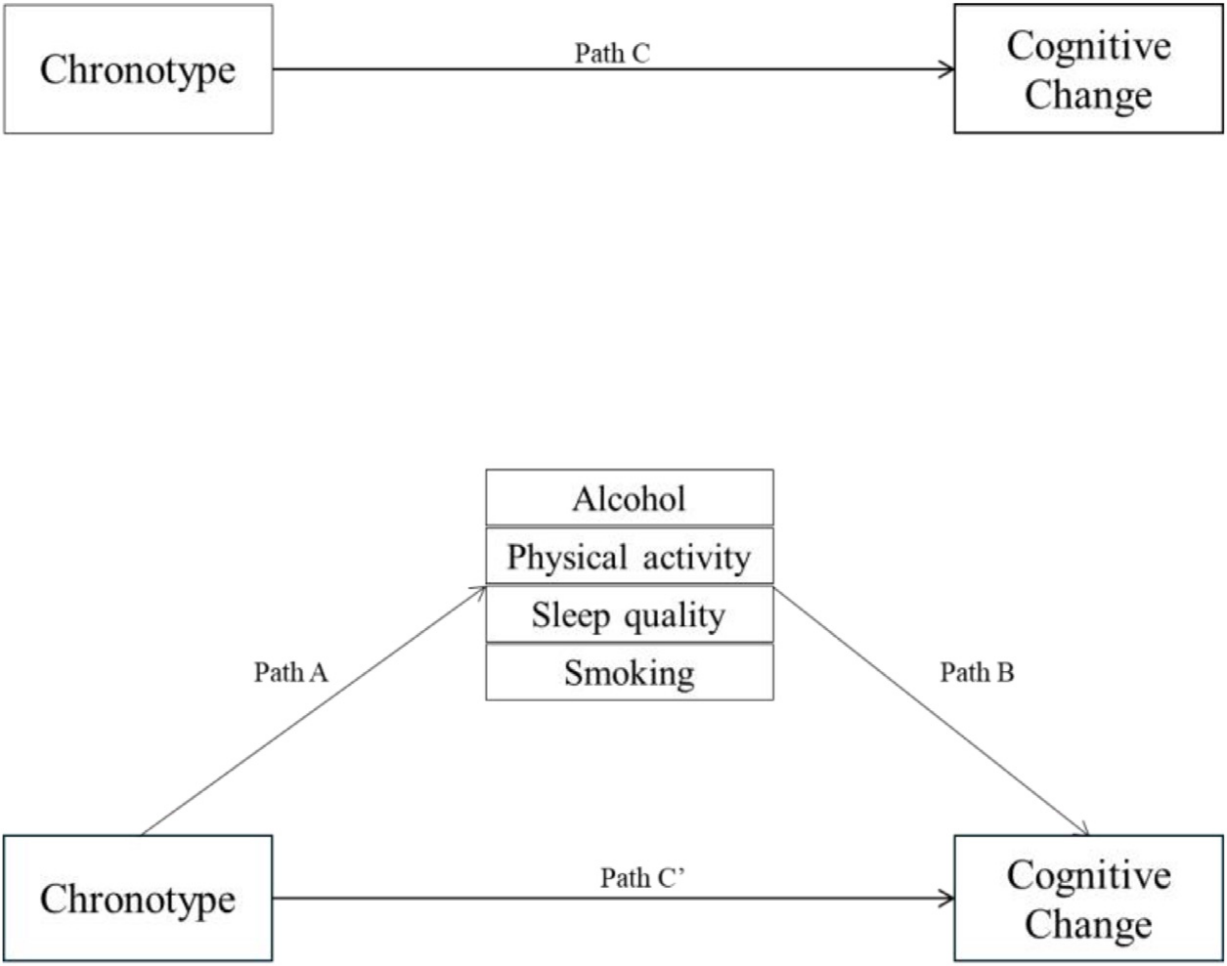
Mediation Analysis Pathways.

Mediation analysis assessed whether alcohol intake (categorical), physical activity (continuous), sleep quality (continuous), and smoking status (categorical) mediated the relationship between chronotype and cognitive decline, using the Karlson-Holm-Breen (KHB) method [46]. The KHB-method was used because it allows for the inclusion of both linear regression (continuous mediators) and multinominal logistic regressions (categorical mediators) within a single model, enabling the calculation of direct and indirect effects with all mediators included simultaneously (Fig. 2). Pathway A presents the association between chronotype and the mediators, adjusted for age, sex, and baseline RFFT score. Pathway B presents the association between the mediators and cognitive change, adjusted for all mediators, age, sex, and baseline RFFT score. Pathway C presents the total association between chronotype and cognitive change, including the effect of the mediators. Pathway C’ presents the direct association between chronotype and cognitive change, excluding the effect of the mediators. Indirect effects through the mediators, along with the percentage of total effect mediated were calculated.

As a sensitivity analysis, the effect of categorising MSF_SC_ was examined. MSF_SC_ is continuous variable with arbitrary category limits, however to assess the robustness of the findings, additional analysis using categories were performed. For this categorisation, cut-off points suggested by Roenneberg et al. [47], which have been derived from several European countries, including the Netherlands, were applied. These cut-offs define early chronotypes as MSFsc ≤3:59, intermediate chronotypes as MSFsc 4:00–4:59, and late chronotypes as MSFsc ≥5:00 [48]. The sensitivity analysis were performed in strata of age, sex and educational attainment if a significant interaction term was observed in the main analysis. Post-hoc analyses were conducted to examine pairwise differences between groups.

Statistical analyses were performed in R, version 4.3.1 and STATA, version 18. The R package mice was used for imputation and the KHB package in STATA was used for mediation analysis.

## 3 Results

The 23,798 participants had a median age of 48.9 years (IQR 44.8 – 50.4), 57.7 % of whom were women. The mean baseline RFFT score was 82.3 points (SD 22.1), and the average chronotype was 3:44 AM (SD 44 min). Table 1 presents the characteristics of the total population and by educational attainment. Participants in the low-educational attainment group were slightly older, had lower scores on the RFFT at baseline and follow-up, were more often current smokers and abstainers, and reported higher physically activity levels compared to the middle- and high-educational attainment group. Participants in the high-educational group were more often male and had a higher proportion of light and heavy alcohol intake. Participants who were lost to follow-up were slightly younger, had a lower educational attainment, had a lower baseline RFFT score, and were more frequently current smokers (Supplementary Table 1). The correlations between all variables are presented in Supplementary Table 2.

**Table 1:** Baseline characteristics of the study population, stratified for educational attainment groups.

	Total population *n* = 23,798	Low-educational*n = 6,822 (28.8 %)	Middle-educational* n = 9,165 (38.5 %)	High-educational*n= 7,811 (32.8 %)	p-value†
Age, years [median (IQR)]	48.9 (44.8–50.4)	50.67 (46.3–58.5)	48.0 (44.2–52.0)	48.7 (44.5–54.5)	<0.001
Sex, female [*n* (%)]	13,742 (57.7 %)	4066 (59.6 %)	5550 (60.6 %)	4126 (52.8 %)	<0.001
Chronotype, midpoint sleep‡ [mean (SD)]	3:44 (0:44)	3:46 (0:46)	3:42 (0:44)	3:46 (0:42)	<0.001
Baseline RFFT score§ [mean (SD)]	82.3 (22.1)	73.4 (21.0)	82.0 (20.8)	90.3 (21.5)	<0.001
Follow-up RFFT score§ [mean (SD)]	60.9 (20.8)	53.5 (20.4)	62.2 (19.8)	65.7 (20.5)	<0.001
Alcohol intake||					<0.001
Abstainers [*n* (%)]	4658 (19.6 %)	1595 (23.4 %)	1951 (21.3 %)	1112 (14.2 %)	..
Light intake [*n* (%)]	17,512 (73.6 %)	4621 (67.7 %)	6608 (72.1 %)	6283 (80.4 %)	..
Heavy intake [*n* (%)]	1628 (6.8 %)	606 (8.9 %)	606 (6.6 %)	416 (5.3 %)	..
Smoking status{					<0.001
Never [*n* (%)]	10,260 (43.1 %)	2339 (34.3 %)	4050 (44.2 %)	3871 (49.6 %)	..
Current [*n* (%)]	3518 (14.8 %)	1255 (18.4 %)	1384 (15.1 %)	879 (11.3 %)	..
Past [*n* (%)]	10,020 (42.1 %)	3228 (47.3 %)	3731 (40.7 %)	3061 (39.2 %)	..
Sleep quality, PSQI score# [median (IQR)]	3.4 (1.7–5.1)	3.4 (2.0–5.1)	3.4 (2.0–5.1)	3.4 (1.7–4.3)	<0.001
Physical activity, hours per week⁎⁎ [median (IQR)]	4.8 (2.0–10.5)	5.5 (2.0–13.5)	5.0 (2.0–12.3)	4.0 (2.0–7.7)	<0.001

⁎ Educational status is based on educational attainment categories from Lifelines^37^. Low educational status includes; no education, primary education, lower or preparatory secondary education and junior general secondary education, middle educational status includes; secondary vocational education, work-based learning pathway, senior general secondary education or pre-university secondary education, and high educational status includes; higher vocational education, university education or higher.

† p-value <0.05 is deemed significant for difference in mean (*t*-test), median (Wilcoxon signed-rank-test) and prevalence (Chi-Square test).

‡ Chronotype is a continuous measure of morningness to eveningness on the Munich Chronotype Questionnaire^36^ and calculated as the point of mid-sleep corrected for sleep debt during working days.

§ Cognition is measured with the score on the Ruff Figural Fluency Test (RFFT)^32^ with a test at baseline and at follow-up.

|| Alcohol intake is categorised as: abstainer (no alcoholic beverages), light alcohol intake (maximum of 3 glasses of alcohol a day on average) and heavy alcohol intake (more than 3 glasses on average a day)^38^.

{ Smoking is categorised as: never (never having smoked for a full year), current (having smoked regularly in the past month) and past (not having smoked regularly in the past month, but having smoked for a full year in the past)^45^.

# Sleep quality was calculated with the Pittsburgh Sleep Quality Index Questionnaire^44^.

⁎⁎ Physical activity is presented as the hours of moderate to vigorous physical activity per week measures with the SQUASH^44^.

### 3.1 Association chronotype and cognitive change

Univariable and multivariable linear regression analyses were performed to assess the association between chronotype and cognitive change over a mean follow-up of 10.77 years (SD 1.55). The data did not show a non-linear relationship between chronotype and cognitive decline. Therefore the quadratic term was excluded from the model and a linear relationship was further tested. In the univariable analysis, a one-hour increase in chronotype was associated with a 0.75-point decline (95 % CI −1.11, −0.39) in the RFFT score over 10 years (Supplementary Table 3). In the multivariable model, adjusted for age and sex and baseline RFFT scores, a one-hour increase in chronotype was associated with a 0.36-point decline (95 % CI −0.65, −0.07) in the RFFT score over 10 years (Table 2). Sensitivity analysis, including categories of chronotype (i.e., early, intermediate and late) revealed that a later chronotype was associated with cognitive decline (Supplementary Table 4 and 5).

**Table 2:** Results of the multivariable linear regression analyses between chronotype and cognitive decline, stratified for educational attainment groups.

Cognitive decline*	Total population *n* = 23,798		Low educational attainment{ *n* = 6,822		Middle educational attainment{ *n* = 9,165		High educational attainment{ *n* = 7,811	
	B (95 % CI)	p-value	B (95 % CI)	p-value	B (95 % CI)	p-value	B (95 % CI)	p-value
Chronotype†	−0.36 (−0.65, −0.07)	0.02	0.07 (−0.44, 0.57)	0.80	−0.41 (−0.88, 0.06)	0.09	−0.80 (−1.34, −0.26)	0.004
Baseline RFFT score‡	−0.63 (−0.64, −0.62)	<0.001	−0.63 (−0.65, −0.61)	<0.001	−0.66 (−0.67, −0.64)	<0.001	−0.66 (−0.68, −0.65)	<0.001
Age§	0.71 (0.35, 1.07)	<0.001	0.70 (0.07, 1.34)	0.03	1.14 (0.51, 1.78)	0.004	0.62 (0.02, 1.23)	0.04
Age-squared§	−0.02 (−0.02, −0.01)	<0.001	−0.02 (−0.02, −0.01)	<0.001	−0.02 (−0.03, −0.01)	<0.001	−0.01 (−0.02, −0.01)	<0.001
Sex (male)	−3.88 (−4.31, −3.45)	<0.001	−4.56 (−5.35, −3.76)	<0.001	−4.09 (−4.80, −3.39)	<0.001	−3.74 (−4.49, −2.99)	<0.001

Explained variance||	Adjusted R-squared		Adjusted R-squared		Adjusted R-squared		Adjusted R-squared	
	0.4052		0.404		0.400		0.412	

⁎ Difference in score on the Ruff Figural Fluency Test (RFFT)^32^ at baseline compared to follow-up after 10 years of follow-up.

† Chronotype is a continuous measure of morningness to eveningness on the Munich Chronotype Questionnaire^36^ and calculated as the point of mid-sleep corrected for sleep debt during working days.

‡ Baseline RFFT score of unique designs^32^.

§ Age and age squared are included as a continuous variable in years.

|| Explained variance is presented as the adjusted R-squared and gives insight in how much of the variance in the data is explained by the included variables with 0 being 0 % is explained and 1 being 100 % explained.

{ Educational status is based on educational attainment categories from Lifelines^37^. Low educational status includes; no education, primary education, lower or preparatory secondary education and junior general secondary education, middle educational status includes; secondary vocational education, work-based learning pathway, senior general secondary education or pre-university secondary education, and high educational status includes; higher vocational education, university education or higher.

Moderation analysis showed that age and sex did not moderate the association between chronotype and cognitive decline, but educational attainment did (*p* = 0.02) (Supplementary Table 6). Stratified analyses revealed a negative association between chronotype and cognitive change among the high-educational attainment group (Table 2). In this group, each one-hour increase in chronotype was associated with a 0.80-point decline (95 % CI −1.34, −0.26) in the RFFT score over 10 years. In the middle-educational attainment group, a borderline significant effect (*p* = 0.09) showed a 0.41-point decline (95 % CI −0.88, 0.06) in the RFFT over 10 years. No association was found in the low-educational group (0.07, 95 % CI −0.44, 0.57). Sensitivity analysis, including categories of chronotype (i.e., early, intermediate and late) revealed that a later chronotype was associated with cognitive decline in the middle- and high-educational attainment group (Supplementary Table 4 and 5).

### 3.2 Mediation analysis by sleep quality and health behaviours

Stratified mediation analysis, as presented in Fig. 2, was performed for the three separate educational attainment groups, examining the mediating roles of sleep quality and health behaviours (alcohol intake, physical activity, and smoking). Stratified mediation analysis was conducted by educational attainment, as a *p* < 0.10 was found for the interaction between chronotype and educational attainment in the multivariable linear regression. Significant total association (path C) and indirect association (path ‘C) were found only in the high-educational attainment group (Supplementary Table 7). The following section shows the results from the high-educational attainment group.

### 3.3 Associations between chronotype and mediators (Path A)

**Fig. 3:**
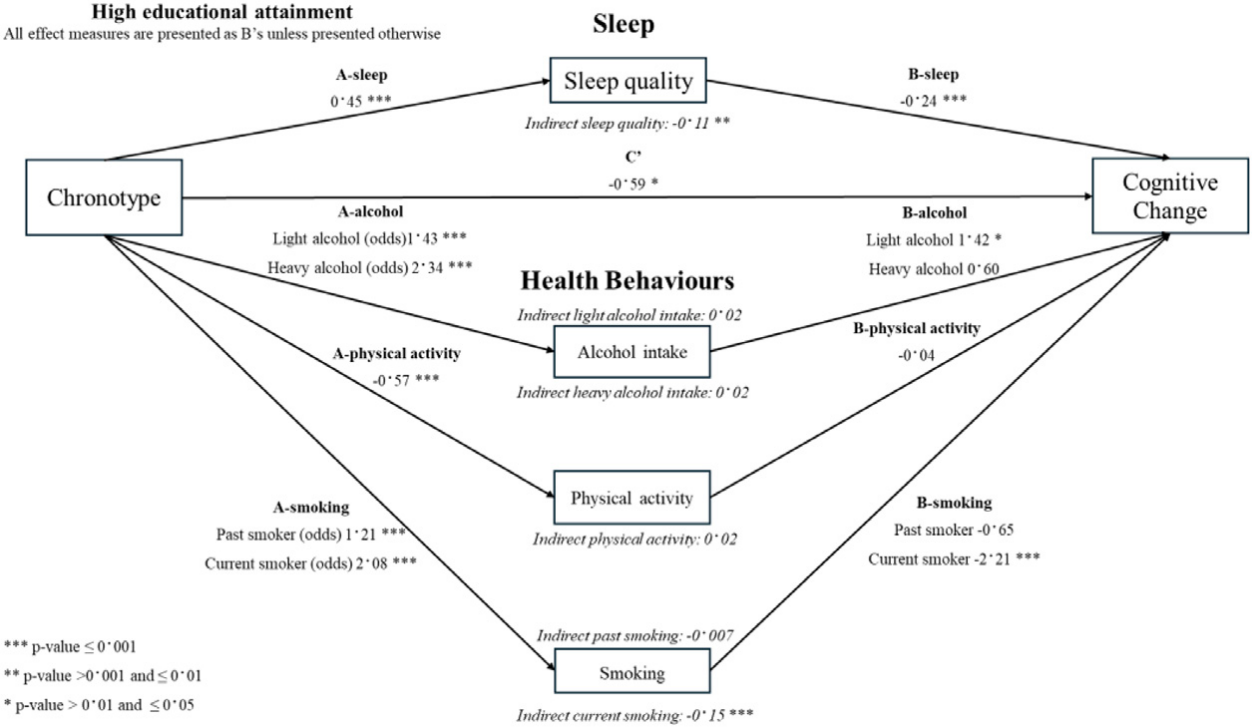
Figural representation of mediation analysis by sleep quality, alcohol intake, physical activity and smoking status.

A one-hour increase in chronotype was associated with a 0.45-point increase (95 % CI 0.37, 0.52) in the PSQI score, reflecting poorer sleep quality (Fig. 3). Additionally, a one-hour increase in chronotype was associated with a 1.43 higher odds (95 % CI 1.34, 1.54) of light alcohol intake and 2.34 higher odds (95 % CI 2.27, 2.41) of heavy alcohol intake compared to abstention. A one-hour increase in chronotype was also associated with 0.57 h decrease (95 % CI −0.82, −0.34) in physical activity per week, and with a 1.21 higher odds (95 % CI 1.14, 1.31) of past smoking and 2.08 higher odds (95 % CI 1.88, 2.29) of current smoking, compared to never smoking.

### 3.4 Associations between sleep quality and health behaviours and cognitive decline (Path B)

A one-point increase on the PSQI, indicating poorer sleep quality, was associated with a 0.24-point decline (95 % CI −0.41, −0.08) in cognition (Fig. 3). Compared to abstainers, light alcohol intake was linked to a 1.42-point increase (95 % CI 0.32, 2.52) in cognition. Current smoking was associated with a 2.21-point decline (95 % CI −3.50, −0.97) in cognition compared to never smokers. No associations were identified between heavy alcohol intake, physical activity, and past smoking and cognitive change.

### 3.5 Direct and indirect association between chronotype and cognitive decline (Path C)

Every one-hour increase in chronotype was associated with 0.80-point decline (95 % CI −1.33, −0.26) in cognition (path C) (Fig. 3). After adjusting for alcohol intake, physical activity, sleep quality, and smoking, the indirect effect remained significant, with a 0.59-point decline (95 % CI −1.14, −0.04) in cognition (path C’). The combined indirect effect of all the mediators was significant (B: −0.21 (95 % CI −0.34, −0.07).

### 3.6 Mediation effects by sleep quality and smoking

Two significant indirect effects were observed: the association between chronotype and cognitive decline was mediated by poorer sleep quality and current smoking (Fig. 3). A one-hour increase in chronotype led to poorer sleep quality, which in turn contributed to a 0.11-point decline (95 % CI −0.18, −0.03) in cognition. Sleep quality mediated 13.5 % of the association after accounting for age, sex, baseline RFFT score, and the mediating effect of the other mediators (Supplementary Table 5). Similarly, a one-hour increase in chronotype was linked to current smoking, which was associated with a 0.15-point decline (95 % CI −0.24, −0.06) in cognition. Smoking mediated 18.6 % of the association after accounting for age, sex, baseline RFFT score, and the mediating effect of the other mediators (Supplementary Table 5). No statistically significant indirect effects were observed for alcohol intake, physical activity, and past smoking. The combined mediating effect of all variables accounted for 25.7 % of the total association (Supplementary Table 7).

## 4 Discussion

In our study, we found that a later chronotype was associated with cognitive decline at 10-year follow-up among-middle aged and older adults, but only among the high educated participants. In the high-educational attainment group, poorer sleep quality (13.52 %) and current smoking (18.64 %) partially mediated the association. Physical activity, past smoking, and alcohol consumption did not explain the association between chronotype and cognitive decline. In the low- and middle-educational attainment subgroups, no significant total (path C) and direct (path C’) effects were observed.

Our findings suggests that highly educated participants with a late chronotype experience greater cognitive decline. The current evidence on chronotype and cognitive decline is heterogenous, probably due to differences in study population, chronotype definitions, cognitive measures and follow-up times [[26], [27], [28]]. For example, Suh et al. [27] found a 40 % lower risk of cognitive decline only in individuals with a late chronotype, while Kim et al. [26] reported greater cognitive decline only in those with an early chronotype. Although we explored a potential U-shaped association, we only observed a negative impact of the late chronotype. One reason why we did not find an association between an early chronotype and cognitive decline could be due to the small group of individuals with an extreme early chronotype, which was estimated at 0.11 % in our population ^6^. Although our study provides new evidence on the association between chronotype and cognitive decline, the question whether chronotype itself (i.e., having an early or late chronotype) or the burden of social jetlag plays a role in cognitive decline and dementia remains. Research indicates that the circadian rhythm may play a key role in regulating the expression of neuroprotective proteins and prevent cerebral oxidative stress [17], but also in blood-brain barrier permeability, which has implications for protein clearance from the brain [21]. Besides this, the circadian rhythm may also modulate immune responses. Human studies investigating these mechanisms are scarce, but animal studies showed that deletion of circadian clock related genes leads to widespread astrocyte activation and synaptic degeneration [49]. These findings highlight the potential importance of circadian rhythms in maintaining immune homeostasis in the brain. Taken together, these potential mechanisms underscore the possibility that chronotype itself could play an independent role in cognitive decline. However, the role of chronotype in cognitive decline may not be entirely independent, as circadian misalignment could also contribute to this association.

If an individual can live according to their own biological clock, the chronotype will reflect the circadian rhythm. However, in case of circadian misalignment leading to social jetlag, this factor could also partially explain the association, rather than chronotype itself [4]. While the MCTQ can measure social jetlag, the simplicity of the calculation has been criticised [6]. Besides this, as indicated by Beauvalet et al., (2017) in their systematic review, there is a large variation in the measurement tools, the computation and the definition of social jetlag [50]. Social jetlag occurs when individuals need to wake up earlier than their biological clock due to, for example, work. This may explain why we did not find an association between chronotype and cognitive decline in the low- and middle-educational attainment groups. These individuals may have more opportunities to find jobs with flexible working hours (e.g., morning construction work, daytime customer service and nighttime bartending) [51] aligning with their chronotype. In contrast, higher-educated individuals often hold positions with rigid 9-to-5 schedules (e.g., executive, manager or teacher), limiting their ability to align work hours with their chronotype [51]. Future research should aim to refine methods for measuring social jetlag, allowing it to be included as a covariate. This would help accounting for circadian misalignment and provide a more accurate estimate of the true association between chronotype and cognitive decline, independent of social jetlag. Another potential explanation for why the observed association was evident only in the high-educational attainment group and not in the other two educational attainment groups, is the possibility of attrition bias. In the low- and middle-educational attainment groups, a larger percentage did not complete the follow-up RFFT assessment. Those who were lost to follow-up had lower baseline cognitive function and therefore may have discontinued participation due to cognitive difficulties. In our study, individuals with higher baseline cognition had more potential to show cognitive decline over time, whereas those with lower baseline cognition may have dropped out before follow-up, as participation in the study and cognitive testing became increasingly challenging. As a result, the overall decline in cognition observed in the low- and middle-educational attainment groups may have been smaller than if those lost to follow-up had remained in the study.

A later chronotype has been linked to lifestyle behaviours such as alcohol intake [10], physical inactivity [12], smoking [11], and poor sleep quality [9], which are themselves established risk factors for cognitive decline [1]. It can be hypothesised that the mediation pathways were likely due to the created social jetlag. In our study, we found that sleep quality and current smoking mediated the association between chronotype and cognitive decline among middle aged and older adults. Approximately 25 % of the association between chronotype and cognitive decline was mediated by poor sleep quality and current smoking.

Individuals with a later chronotype often have poorer sleep quality, including shorter sleep duration and more disturbances [9]. Research shows that short sleep duration is linked to brain volume loss (e.g., grey matter and hippocampus) [52] and disruptions in rapid-eye-movement (REM) sleep [53]. Disruption of the REM sleep could impair the quality and continuity of the non-REM sleep phase, and thereby cause fragmented non-REM sleep [54]. During the non-REM phase, the brain clears metabolic byproducts and waste [53] such as Aβ, of which its accumulation is a known risk factor for dementia [1]. Though evidence suggests that sleep quality influences cognitive decline, longitudinal studies remain scare with only two prior studies finding an association between poor sleep quality and cognitive decline [55,56]. Due to the lack of robust evidence, the Lancet Dementia Commission has emphasised the need for further research into the effect of sleep variables in their 2024 Dementia Prevention Report [1]. Our study is the first examining the mediating role of sleep quality in the association between chronotype and cognitive decline and thereby extends previous findings showing that poor sleep quality mediates the association between chronotype and mental health problems [57], and depressive symptoms [58]. In addition to the health behaviours and sleep quality, also depression and social contact could be mediating factors in the association between chronotype and cognitive decline. Depression and social contact are risk factors for cognitive decline [1] and poor sleep quality [59], but chronotype is also associated with depression and social contact [60]. This highlights the complexity of the pathways in the association between chronotype and cognitive decline.

We found that the association between chronotype and cognitive decline was also mediated by current smoking. Prior studies suggested that a late chronotype is associated with an increased risk of smoking [11]. Smoking may potentially serve as a coping mechanism for social jetlag due to nicotine's short-term cognitive-enhancing effects [61]. Additionally, social jetlag is also associated with higher stress, which in turn is associated with a higher risk of smoking [11]. Chronic smoking has been associated with accelerated brain ageing and with white matter degeneration [62], resulting in cognitive decline. While no studies have yet examined smoking as a mediator between chronotype and cognitive decline, evidence suggests that smoking mediates the association between chronotype and psychological well-being [63] and depressive symptoms [64]. As research suggests a bidirectional association between smoking and sleep quality [11], it further underscores the complex interplay of multiple risk factors contributing to cognitive decline.

We found that for every one-hour increase in chronotype, cognition declined by 0.80 points among the high-educational attainment group over a 10-year follow-up. With a maximum 9 h range between the minimum and maximum chronotype, this translates to a potential 7.2-point decline on the RFFT for the most extreme late chronotypes. This corresponds to a small-to-moderate standardised effect size (0.36). As this measure assesses non-verbal fluency and executive function, we cannot comment on the impact of chronotype on other cognitive domains. Additionally, given our 10-year follow-up, the decline could be greater over longer periods (e.g., from 40 to 70 years old). Notably, a quarter of the association between chronotype and cognitive decline was mediated by sleep quality and health behaviours, emphasising the need for preventive interventions addressing both chronotype-related social jetlag and lifestyle factors.

One strength of our study is the relatively long follow-up period and large sample size, allowing for stratified analysis by educational attainment. The Lifelines cohort is representative of the Dutch population, and the MSF_SC_ is regarded as one of the most reliable subjective measures of chronotype [6]. However, several limitations should be acknowledged. Firstly, attrition bias may have resulted in underestimating the association, particularly in the low-educational attainment group, where nearly half of the participants lost to follow-up came from. These individuals had lower baseline cognitive function, and may have dropped out due to cognitive issues, suggesting that the true cognitive decline in this group could be greater than observed. Future studies using registry-based dementia diagnosis could address this issue. Additionally, the RFFT was only measured twice. More frequent measurement would have allowed for a more precise analysis of cognitive decline trajectories. Also, since late chronotypes tend to perform worse in the morning, the lack of data on the timing of RFFT administration could influence the reliability. Future studies should record both the date and time of cognitive test administration to correct for this potential bias. Lastly, this study focussed on health behaviours as mediating factors. However, both mental and physical diseases might also mediate this association, such as depressive symptoms, diabetes, hypertension or cardiovascular diseases [1]. Examining their mediating role is an interesting avenue for future research.

To conclude chronotype was associated with cognitive decline among middle aged and older highly educated participants. The significant indirect effects revealed that 13.2 % and 16.8 % of this association was mediated by sleep quality and current smoking, respectively. The mediating effects highlight the importance of targeted preventive measures, such as smoking cessation programmes, and the promotion of flexible work schedules, particularly among highly educated individuals.

## Funding

This work is part of the BIRD-NL consortium funded by the Dutch Medical Research Council (ZonMw) as part of the National Dementia Strategy 2021–2030 by the Dutch Ministry of Health, Welfare and Sport (grant number: 1051003210005).

## Data sharing

Data may be obtained from a third party and are not publicly available. Researchers can apply to use the Lifelines data used in this study. More information about how to request Lifelines data and the conditions of use can be found on their website (http://www.lifelines-biobank.com). Data for the current project can be obtained under the mention of project number: OV23_00871.

## CRediT authorship contribution statement

**A.N. Wenzler:** Writing – review & editing, Writing – original draft, Software, Formal analysis, Conceptualization. **A.C. Liefbroer:** Writing – review & editing, Supervision, Methodology, Conceptualization. **R.C. Oude Voshaar:** Writing – review & editing, Supervision, Methodology, Conceptualization. **N. Smidt:** Writing – review & editing, Supervision, Methodology, Conceptualization.

## Declaration of competing interest

We declare no conflict of interest.

## Appendix Supplementary materials

Image, application 1
