# Supplementary material for: Chronotype as a potential risk factor for cognitive decline: The mediating role of sleep quality and health behaviours in a 10-year follow-up study
Source: J Prev Alzheimers Dis. 2025 Apr 11;12(6):100168. doi: 10.1016/j.tjpad.2025.100168 (PMC12434255; doi:10.1016/j.tjpad.2025.100168)
Supplement: Supplementary file 1 [file mmc1.docx]

**Supplementary materials**

# Supplementary Table 1. Baseline characteristics of the population for analysis and the population loss to follow-up

| **Variable** | **Population for analysis**^*^ ***n* = 23,798** | **Loss to follow-up**^*^  ***n*=21,127** | **p-value**^†^ |
| --- | --- | --- | --- |
| Age, years [median (IQR)] | 48.9 (44.8 – 50.4) | 50.0 (45.2 – 60.42) | <0.001 |
| Sex, female [*n* (%)] | 13,742 (57.7%) | 12,413 (58.8%) |  |
| Educational attainment^‡^ |  |  | <0.001 |
| Low [*n* (%)] | 6,822 (28.7%) | 9,347 (44.2%) | .. |
| Middle [*n* (%)] | 9,165 (38.5%) | 7,260 (34.4%) | .. |
| High [*n* (%)] | 7,811 (32.8%) | 4,520 (21.4%) | .. |
| Chronotype^§^ [mean (SD)] | 3:44 (0:44) | 3:46 (0:47) | 0.82 |
| RFFT baseline ^\|\|^ [mean (SD)] | 82.3 (22.1) | 74.87 (23.36) | <0.001 |
| RFFT follow-up ^\|\|^ [mean (SD)] | 60.9 (20.8) | .. | .. |
| Alcohol intake^{^ |  |  | <0.001 |
| Abstainers [*n* (%)] | 4,658 (19.6%) | 4,877 (23.1%) | .. |
| Light intake [*n* (%)] | 10,672 (44.8%) | 9,003 (42.6%) | .. |
| Heavy intake [*n* (%)] | 8,468 (35.6%) | 7,247 (34.3%) | .. |
| Smoking status*^#^* |  |  | <0.001 |
| Never [*n* (%)] | 10,260 (43.1%) | 8,219 (38.9%) | .. |
| Current [*n* (%)] | 3,518 (14.8%) | 4,230 (20.0%) | .. |
| Past [*n* (%)] | 10,020 (42.1%) | 8,678 (41.1%) | .. |
| Sleep quality^**^ [median (IQR)] | 3.4 (1.7 – 5.1) | 3.4 (2.0 – 5.1) | <0.001 |
| Physical activity^††^ [median (IQR)] | 4.8 (2.0 – 10.5) | 4.7 (1.8 – 11.0) | <0.001 |

^*^ Population for analysis are included in the main analysis, loss to follow-up population was lost due no information on the RFFT-test at follow-up.

^†^ p-value <0.05 is deemed significant for difference in mean (t-test), median (Wilcoxon signed-rank-test) and prevalence (Chi-Square test)

^‡^ Educational status is based on educational attainment categories from Lifelines [37]. Low educational status includes; no education, primary education, lower or preparatory secondary education and junior general secondary education, middle educational status includes; secondary vocational education, work-based learning pathway, senior general secondary education or pre-university secondary education, and high educational status includes; higher vocational education, university education or higher

^§^ Chronotype is a continuous measure of morningness to eveningness on the Munich Chronotype Questionnaire [36] and calculated as the point of mid-sleep corrected for sleep debt during working days

^||^ Cognition is measures with the score on the Ruff Figural Fluency Test (RFFT) [32] with a test at baseline and at follow-up

^{^ Alcohol intake is categorised as: abstainer (no alcoholic beverages), light alcohol intake (maximum of 3 glasses of alcohol a day on average) and heavy alcohol intake (more than 3 glasses on average a day) [38]

*^#^* Smoking is categorised as: never (never having smoked for a full year), current (having smoked regularly in the past month) and past (not having smoked regularly in the past month, but having smoked for a full year in the past) [45]

^**^ Sleep quality was calculated with the Pittsburgh Sleep Quality Index Questionnaire [44]

^††^ Physical activity is presented as hours of moderate to vigorous physical activity per week measures with the SQUASH [41]

**Supplementary Table 2.** Correlations between the different variables included in the study

|  | Chronotype^*^ | Cognitive change^†^ | Baseline RFFT score^‡^ | Age^§^ | Sex^\|\|^ | Educational attainment^{^ | Sleep quality^#^ | Alcohol intake^**^ | Physical activity^††^ | Smoking^‡‡^ |
| --- | --- | --- | --- | --- | --- | --- | --- | --- | --- | --- |
| Chronotype^*^ | 1.00 | -0.04 | 0.02 | 0.07 | -0.02 | 0.01 | 0.15 | 0.12 | -0.07 | 0.14 |
| Cognitive  change^†^ | -0.04 | 1.00 | -0.55 | -0.15 | -0.08 | -0.09 | 0.01 | -0.05 | 0.02 | -0.03 |
| Baseline  RFFT score^‡^ | 0.02 | -0.55 | 1.00 | -0.26 | -0.05 | 0.30 | -0.03 | 0.02 | -0.08 | -0.04 |
| Age^§^ | 0.07 | -0.15 | -0.26 | 1.00 | 0.06 | -0.11 | 0.03 | 0.07 | -0.05 | 0.07 |
| Sex^\|\|^ | -0.2 | -0.08 | -0.05 | 0.06 | 1.00 | 0.06 | -0.18 | 0.29 | 0.13 | 0.02 |
| Educational attainment^{^ | 0.01 | -0.09 | 0.30 | -0.11 | 0.06 | 1.00 | -0.05 | 0.05 | -0.15 | -0.12 |
| Sleep  quality^#^ | 0.15 | 0.01 | -0.03 | 0.03 | -0.18 | -0.05 | 1.00 | -0.06 | -0.03 | 0.04 |
| Alcohol  intake^**^ | 0.12 | -0.05 | 0.02 | 0.07 | 0.29 | 0.05 | -0.06 | 1.00 | 0.06 | 0.14 |
| Physical  activity^††^ | -0.07 | 0.02 | -0.08 | -0.05 | 0.13 | -0.15 | -0.03 | 0.06 | 1.00 | 0.02 |
| Smoking^‡‡^ | 0.14 | -0.03 | -0.04 | 0.07 | 0.02 | -0.12 | 0.02 | 0.14 | 0.02 | 1.00 |

^*^ Chronotype is a continuous measure of morningness to eveningness on the Munich Chronotype Questionnaire [36] and calculated as the point of mid-sleep corrected for sleep debt during working days

^†^ Difference in score on the Ruff Figural Fluency Test (RFFT) at baseline compared to follow-up after 10 years of follow-up

^‡^ Baseline RFFT score of unique designs [32]

^§^ Age is a continuous variable in years

^||^ Sex is coded as 0 being female, and 1 being male

^{^ Educational status is based on educational attainment categories from Lifelines [37]. Low educational status (coded as 0) includes; no education, primary education, lower or preparatory secondary education and junior general secondary education, middle educational status (coded as 1) includes; secondary vocational education, work-based learning pathway, senior general secondary education or pre-university secondary education, and high educational status (coded as 2) includes; higher vocational education, university education or higher

^#^ Sleep quality was calculated with the Pittsburgh Sleep Quality Index Questionnaire [44]

^**^ Alcohol intake is categorised as: abstainer (no alcoholic beverages) coded as 0, light alcohol intake (maximum of 3 glasses of alcohol a day on average) coded as 1, and heavy alcohol intake (more than 3 glasses on average a day) coded as 2 [38]

^††^ Smoking is categorised as: never (never having smoked for a full month) coded as 0, current (having smoked regularly in the past month) coded as 1, and past (not having smoked regularly in the past month, but having smoked for a full month in the past) coded as 2 [45]

**Supplementary Table 3.** Uni- and multivariable linear regression between chronotype and cognitive change

| Cognitive decline^*^  n=23,798 | Univariable regression^{^ | |
| --- | --- | --- |
|  | B (95% CI) | p-value |
| Chronotype^†^ | -0.75 (-1.11, -0.39) | <0.001 |
| Baseline RFFT score^‡^ | -0.54 (-0.55, -0.53) | <0.001 |
| Age^§^ | 0.63 (0.17, 1.09) | 0.01 |
| Age squared^§^ | -0.01 (-0.01, -0.01) | <0.001 |
| Sex (male) | -5.47 (-6.00, -4.94) | <0.001 |

^*^ Difference in score on the Ruff Figural Fluency Test (RFFT) [32] at baseline compared to follow-up after 10 years of follow-up

^†^ Chronotype is a continuous measure of morningness to eveningness on the Munich Chronotype Questionnaire [36] and calculated as the point of mid-sleep corrected for sleep debt during working days

^‡^ Baseline RFFT score of unique designs [32]

^§^ Age and age squared are added to the model together and included as a continuous variable in years

^||^ Explained variance is presented as the adjusted R-squared and gives insight in how much of the variance in the data is explained by the included variables with 0 being 0% is explained and 1 being 100% explained. Adjusted R-squared is not presented for the univariable regression coefficients

^{^ Univariable regression was performed for all the variables included in the final multivariable regression model to investigate the effect of all the variables separately on the outcome cognitive decline

**Supplementary Table 4.** Results of the multivariable linear regression analyses between chronotype categorised as early, intermediate and late and cognitive decline

| Cognitive decline^*^ | Total population  *n* = 23,798 | | Low educational attainment^{^  *n* = 6,822 | | Middle educational attainment^{^  *n* = 9,165 | | High educational attainment^{^  *n* = 7,811 | |
| --- | --- | --- | --- | --- | --- | --- | --- | --- |
|  | B (95% CI) | p-value | B (95% CI) | p-value | B (95% CI) | p-value | B (95% CI) | p-value |
| Early chronotype^†^ | Ref | Ref | Ref | Ref | Ref | Ref | Ref | Ref |
| Intermediate chronotype^†^ | -0.25 (-0.69, 0.19) | 0.27 | 0.31 (0.50, 1.12) | 0.45 | -0.49 (-1.19, 0.22) | 0.18 | -0.49 (-1.26, 0.28) | 0.22 |
| Late chronotype^†^ | -1.75 (-2.76, -0.74) | <0.001 | -0.18 (-1.92, 1.56) | 0.84 | -2.35 (-4.05, -0.65) | 0.01 | -2.32 (-4.11, -0.54) | 0.01 |
| Baseline RFFT score^‡^ | -0.63 (-0.64, -0.62) | <0.001 | -0.63 (-0.65, -0.61) | <0.001 | -0.66 (-0.67, -0.64) | <0.001 | -0.66 (-0.68, -0.65) | <0.001 |
| Age^§^ | 0.72 (0.36, 1.08) | <0.001 | 0.70 (0.07, 1.34) | 0.03 | 1.16 (0.52, 1.80) | <0.001 | 0.61 (0.01, 1.22) | 0.05 |
| Age-squared^§^ | -0.02 (-0.02, -0.01) | <0.001 | -0.01 (-0.02, -0.01) | <0.001 | -0.02 (-0.03, -0.01) | <0.001 | -0.01 (-0.02, -0.01) | <0.001 |
| Sex (male) | -3.88 (-4.31, -3.45) | <0.001 | -4.55 (-5.35, -3.76) | <0.001 | -4.09 (-4.80, -3.38) | <0.001 | -3.75 (-4.50, -3.00) | <0.001 |
|  | | | | | | | | |
| Explained variance^\|\|^ | Adjusted R-squared | | Adjusted R-squared | | Adjusted R-squared | | Adjusted R-squared | |
|  | 0.41 | | 0.40 | | 0.40 | | 0.41 | |

^*^ Difference in score on the Ruff Figural Fluency Test (RFFT) [32] at baseline compared to follow-up after 10 years of follow-up

^†^ Chronotype is a categorised variant mid-sleep corrected for sleep debt during working days^36^ based on cut-off points suggested by Roenneberg et al., [48]: early chronotype MSFsc ≤3:59, intermediate chronotype MSFsc 4:00-4:59 and late chronotypes MSFsc ≥5:00

^‡^ Baseline RFFT score of unique designs [32]

^§^ Age and age squared are included as a continuous variable in years

^||^ Explained variance is presented as the adjusted R-squared and gives insight in how much of the variance in the data is explained by the included variables with 0 being 0% is explained and 1 being 100% explained.

^{^ Educational status is based on educational attainment categories from Lifelines [37]. Low educational status includes; no education, primary education, lower or preparatory secondary education and junior general secondary education, middle educational status includes; secondary vocational education, work-based learning pathway, senior general secondary education or pre-university secondary education, and high educational status includes; higher vocational education, university education or higher

**Supplementary Table 5.** Post-Hoc comparison of chronotype categories across educational attainment groups

| Comparison chronotypes^*^ | Total population | | Low educational attainment^†^ | | Middle educational attainment^†^ | | High educational attainment^†^ | |
| --- | --- | --- | --- | --- | --- | --- | --- | --- |
|  | Population size | p-value | Population size | p-value | Population size | p-value | Population size | p-value |
| Early (0) vs. intermediate (1) | *n* = 10,350 | 0.27 | *n* = 2,869 | 0.45 | *n* = 4,218 | 0.18 | *n* = 3,263 | 0.22 |
| Early (0) vs. late (2) | *n* = 12,270 | <0.001 | *n* = 3,564 | 0.84 | *n* = 4,536 | 0.01 | *n* = 4,170 | 0.01 |
| Intermediate (1) vs. late (2) | *n* = 1,178 | 0.003 | *n* = 389 | 0.58 | *n* = 411 | 0.03 | *n* = 378 | 0.04 |

^*^ Chronotype is a categorised variant mid-sleep corrected for sleep debt during working days [36] based on cut-off points suggested by Roenneberg et al., [48] : early chronotype MSFsc ≤3:59, intermediate chronotype MSFsc 4:00-4:59 and late chronotypes MSFsc ≥5:00

^†^ Educational status is based on educational attainment categories from Lifelines [37]. Low educational status includes; no education, primary education, lower or preparatory secondary education and junior general secondary education, middle educational status includes; secondary vocational education, work-based learning pathway, senior general secondary education or pre-university secondary education, and high educational status includes; higher vocational education, university education or higher

# Supplementary Table 6. Interaction analysis between the previous models and the moderators (age, sex and educational attainment)

| **Variable** | **Interaction term** | **p-value**^‡^ |
| --- | --- | --- |
| Cognitive Decline ~ Chronotype * Sex + 1A + Age | | |
| Sex (male) | Chronotype * Sex | 0.74 |
| Cognitive Decline ~ Chronotype * Age + 1A + Sex | | |
| Age continuous | Chronotype * Age | 0.89 |
| Age categorical^*^ | Chronotype * older adult | 0.41 |
| Cognitive Decline ~ Chronotype * education + 1A + Sex + Age | | |
| Education^†^ | Chronotype * middle | 0.14 |
|  | Chronotype * high | 0.02 |

^*^ Age was included in two categories: middle-aged adults (40-55 years old) and older adults (55+ years old)

^†^ Educational status is based on educational attainment categories from Lifelines [37]. Low educational status includes; no education, primary education, lower or preparatory secondary education and junior general secondary education, middle educational status includes; secondary vocational education, work-based learning pathway, senior general secondary education or pre-university secondary education, and high educational status includes; higher vocational education, university education or higher

^‡^ A p-value of <0.10 is deemed significant in the interaction analysis

# Supplementary Table 7. Mediation analysis by sleep quality and health behaviours in three educational attainment groups

| **Mediation analysis association chronotype**^*^ **and cognitive decline**^†^**^,^**^‡^ | **Total population**  **n = 23,798** | | **Low educational attainment**^§^  **n = 6,822** | | **Middle educational attainment**^§^  **n = 9,165** | | **High educational attainment**^§^  ***n* = 7,811** | |
| --- | --- | --- | --- | --- | --- | --- | --- | --- |
| Sleep quality^\|\|^ | | | | | | | | |
|  | Effect estimate (95% CI) | p | Effect estimate (95% CI) | p | Effect estimate (95% CI) | p | Effect estimate (95% CI) | p |
| Path A. (*Chronotype to sleep quality*) | 0.48 (0.44, 0.52) | <0.001 | 0.51 (0.43, 0.58) | <0.001 | 0.48 (0.41, 0.54) | <0.001 | 0.45 (0.37, 0.52) | <0.001 |
| Path B. (*Sleep quality to cognitive change*) | -0.18 (-0.27, -0.09) | <0.001 | -0.10 (-0.26, 0.05) | 0.19 | -0.17 (-0.31, -0.03) | 0.02 | -0.24 (-0.41, -0.08) | 0.004 |
| Indirect (*Chronotype through sleep quality to cognitive change*) | -0.09 (-0.13, -0.04) | <0.001 | -0.05 (-0.13, 0.03) | 0.19 | -0.08 (-0.15, -0.01) | 0.02 | -0.11 (-0.18, -0.03) | 0.01 |
|  | | | | | | | | |
| Health behaviours | | | | | | | | |
| Path A. (*Chronotype to Mediators*) | Effect estimate (95% CI) | p | Effect estimate (95% CI) | p | Effect estimate (95% CI) | p | Effect estimate (95% CI) | p |
| Alcohol intake^{^ (*reference is abstainer*) | Ref | Ref | Ref | Ref | Ref | Ref | Ref | Ref |
| Light intake (odds) | 1.36 (1.30, 1.42) | <0.001 | 1.27 (1.17, 1.38) | <0.001 | 1.41 (1.31, 1.52) | <0.001 | 1.43 (1.34, 1.54) | <0.001 |
| Heavy intake (odds) | 2.14 (1.97, 2.29) | <0.001 | 1.90 (1.68, 2.16) | <0.001 | 2.32 (2.03, 2.61) | <0.001 | 2.34 (2.27, 2.41) | <0.001 |
| Physical activity*^#^* (minutes moderate to vigorous per week) | -0.89 (-1.08, 0.70) | <0.001 | -0.54 (-0.92, -0.15) | 0.006 | -1.17 (-1.51, -0.84) | <0.001 | -0.57 (0.82, 0.34) | <0.001 |
| Smoking status^**^ (*reference is never smoking*) | Ref | Ref | Ref | Ref | Ref | Ref | Ref | Ref |
| Past smoker (odds) | 1.17 (1.14, 1.22) | <0.001 | 1.13 (1.05, 1.22) | <0.001 | 1.21 (1.13, 1.28) | <0.001 | 1.21 (1.14, 1.31) | <0.001 |
| Current smoker (odds) | 1.84 (1.75, 1.94) | <0.001 | 1.60 (1.46, 1.75) | <0.001 | 1.94 (1.79, 2.12) | <0.001 | 2.08 (1.88, 2.29) | <0.001 |
| Path B. (*Mediators to Cognitive change*) | | | | | | | | |
| Alcohol intake^{^ (*reference is abstainer*) | Ref | Ref | Ref | Ref | Ref | Ref | Ref | Ref |
| Light intake | 1.61 (1.05, 2.17) | <0.001 | 1.87 (0.90, 2.84) | <0.001 | 0.68 (-0.19, 1.57) | 0.34 | 1.42 (0.32, 2.52) | 0.01 |
| Heavy intake | 0.88 (-0.12, 1.88) | 0.09 | 2.06 (0.90, 3.72) | 0.01 | 0.20 (-1.43, 1.83) | 0.81 | 0.60 (-1.37, 2.57) | 0.55 |
| Physical activity*^#^* (minutes moderate to vigorous per week) | -0.06 (-0.08, -0.04) | <0.001 | -0.05 (-0.08, -0.01) | 0.01 | -0.05 (-0.07, -0.02) | 0.003 | -0.04 (-0.09, 0.01) | 0.14 |
| Smoking status^**^ (*reference is never smoking*) | Ref | Ref | Ref | Ref | Ref | Ref | Ref | Ref |
| Past smoker | -0.53 (-1.00, -0.05) | 0.03 | 0.42 (-0.47, 1.30) | 0.36 | -0.39 (-1.16, 0.38) | 0.32 | -0.65 (-1.48, 0.17) | 0.12 |
| Current smoker | -2.60 (-3.26, -1.95) | <0.001 | -1.84 (-2.98, -0.70) | 0.002 | -2.22 (-3.27, -1.17) | <0.001 | -2.21 (-3.46, -0.95) | 0.001 |
| Indirect (*Chronotype through Mediators to Cognitive change*) | | | | | | | | |
| Alcohol intake^{^ (*reference is abstainer*) | Ref | Ref | Ref | Ref | Ref | Ref | Ref | Ref |
| Light intake | 0.02 (0.009, 0.04) | 0.001 | 0.01 (-0.01, 0.04) | 0.30 | 0.02 (-0.01, 0.04) | 0.16 | 0.02 (-0.00, 0.04) | 0.09 |
| Heavy intake | 0.03 (-0.00, 0.06) | 0.09 | 0.08 (0.01, 0.14) | 0.02 | 0.01 (-0.05, 0.06) | 0.81 | 0.02 (-0.04, 0.07) | 0.55 |
| Physical activity*^#^* (minutes moderate to vigorous per week) | 0.06 (0.04, 0.08) | <0.001 | 0.02 (0.00, 0.05) | 0.05 | 0.05 (0.02, 0.09) | 0.01 | 0.02 (-0.01, 0.05) | 0.16 |
| Smoking status^**^ (*reference is never smoking*) | Ref | Ref | Ref | Ref | Ref | Ref | Ref | Ref |
| Past smoker | 0.00 (-0.00, 0.01) | 0.87 | -0.00 (-0.02, 0.01) | 0.45 | -0.00 (-0.01, 0.01) | 0.89 | -0.01 (-0.02, 0.01) | 0.32 |
| Current smoker | -0.18 (-0.23, -0.13) | <0.001 | -0.11 (-0.18, -0.04) | 0.003 | -0.17 (-0.25, -0.09) | <0.001 | -0.15 (-0.24, -0.06) | <0.001 |
|  | | | | | | | | |
| Combined mediation | Beta (95% CI) | P |  |  |  |  | Beta (95% CI) | P |
| Path C. (*Chronotype to cognitive change*) | -0.36 (-0.65, -0.07) | 0.02 | 0.06 (-0.44, 0.57) | 0.80 | -0.41 (-0.88, 0.06) | 0.09 | -0.80 (-1.33, -0.26) | 0.003 |
| Total indirect effect (*Chronotype through mediators to cognitive change*) | -0.16 (-0.23, -0.08) | <0.001 | -0.05 (-0.18, 0.78) | 0.40 | -0.17 (-0.30, -0.05) | 0.01 | -0.21 (-0.34, -0.07) | 0.002 |
| Path C’. (*Chronotype to cognitive change without effect mediators*) | -0.21 (-0.51, 0.10) | 0.18 | 0.12 (-0.40, 0.64) | 0.66 | -0.24 (-0.72, 0.25) | 0.34 | -0.59 (-1.14, -0.04) | 0.03 |
|  | | | | | | | | |
| Percentage mediated by Mediators^††^ | Percentage | p | Percentage | p | Percentage | p | Percentage | p |
| Alcohol intake^{^ (*reference is abstainer*) | Ref | Ref | Ref | Ref | Ref | Ref | Ref | Ref |
| Light intake | -6.79% | 0.001 | 22.50% | 0.30 | -3.74% | 0.16 | -2.54% | 0.09 |
| Heavy intake | -7.86% | 0.09 | 117.48% | 0.02 | -1.70% | 0.81 | -2.13% | 0.55 |
| Physical activity*^#^* (minutes moderate to vigorous per week) | -15.57% | <0.001 | 37.57% | 0.05 | -12.77% | 0.006 | -2.63% | 0.16 |
| Sleep quality^\|\|^ (points on PSQI) | 24.02% | <0.001 | -80.57% | 0.19 | 19.89% | 0.02 | 13.52% | 0.005 |
| Smoking status^**^ (*reference is never smoking*) | Ref | Ref | Ref | Ref | Ref | Ref | Ref | Ref |
| Past smoker | -0.10% | 0.87 | -6.64% | 0.45 | 0.09% | 0.89 | 0.81% | 0.32 |
| Current smoker | 49.26% | <0.001 | -173.04% | 0.003 | 40.70% | <0.001 | 18.64% | <0.001 |
| Total | 42.96% | - | -82.69 | - | 42.27% | - | 25.66% | - |

^*^ Chronotype is a continuous measure of morningness to eveningness on the Munich Chronotype Questionnaire [36] and calculated as the point of mid-sleep corrected for sleep debt during working days

^†^ Cognitive decline was calculated by subtracting the score of unique designs on the RFFT at baseline from the score of unique designs on the RFFT at follow-up [32]

^‡^ All associations have been adjusted for age, sex and baseline cognition (the score on the RFFT at baseline)

^§^ Educational status is based on educational attainment categories from Lifelines [37]. Low educational status includes; no education, primary education, lower or preparatory secondary education and junior general secondary education, middle educational status includes; secondary vocational education, work-based learning pathway, senior general secondary education or pre-university secondary education, and high educational status includes; higher vocational education, university education or higher

^||^ Sleep quality was calculated with the Pittsburgh Sleep Quality Index Questionnaire [44]

^{^ Alcohol intake is categorised as: abstainer (no alcoholic beverages), light alcohol intake (maximum of 3 glasses of alcohol a day on average) and heavy alcohol intake (more than 3 glasses on average a day) [38]

*^#^* Physical activity is presented as the total hours of moderate to vigorous physical activity per week measures with the SQUASH [41]

^**^ Smoking is categorised as: never (never having smoked for a full month), current (having smoked regularly in the past month) and past (not having smoked regularly in the past month, but having smoked for a full month in the past) [45]

^††^ Percentage mediated is calculated by dividing the indirect effect by the total effect. Due to the non-significant total and direct association in the low- and middle-educational attainment group, presented percentages cannot be interpreted as they are an overestimation of the true mediation.
